# Rethinking the 8th AJCC System: Is It Suitable for Patients Aged <55 Years With Stage T4N1M0 Follicular Variant of Papillary Thyroid Carcinoma to Be Placed in Stage I?

**DOI:** 10.3389/fonc.2020.543055

**Published:** 2020-12-11

**Authors:** Wei Long, Di Hu, Ling Zhou, Yueye Huang, Wen Zeng, Sichao Chen, Yihui Huang, Man Li, Min Wang, Wei Zhou, Jianglong Huang, Wei Wei, Chao Zhang, Zeming Liu, Liang Guo

**Affiliations:** ^1^ Department of Plastic Surgery, Zhongnan Hospital of Wuhan University, Wuhan, China; ^2^ Department of Endocrinology and Metabolism and the Shanghai Research Center of Thyroid Diseases, The Shanghai Tenth People’s Hospital, Tongji University School of Medicine, Shanghai, China; ^3^ Department of Ophthalmology, Zhongnan Hospital of Wuhan University, Wuhan, China; ^4^ Department of Pediatrics, St John Hospital and Medical Center, Detroit, MI, United States; ^5^ Department of Cardiovascular Surgery, Union Hospital, Tongji Medical College, Huazhong University of Science and Technology, Wuhan, China

**Keywords:** prognosis, survival, SEER, follicular papillary thyroid carcinoma, TNM staging, AJCC system

## Abstract

**Purpose:**

The newest (8th) edition of the TNM staging system published in 2017. In this edition, some significant changes happened from the previous edition. As a result, down-staging appeared in nearly one third of DTC patients. However, we don’t know whether the new system predicts the survival of FVPTC patients accurately. Therefore, it is necessary to thoroughly evaluate the correlation between the new system and survival prediction in terms of FVPTC.

**Methods:**

We enrolled 17,662 FVPTC patients from the Surveillance, Epidemiology, and End Results database. Factors associated with survival were identified by Cox regression analyses. The mortality rates per 1,000 person-years were calculated and compared. Cox proportional hazards regression quantified the risk of survival, and survival curves were produced by Kaplan-Meier analyses using log-rank tests.

**Results:**

Age at diagnosis, race, T-stage at diagnosis, distant metastasis, radiation therapy, and surgery were independent factors associated with cancer-specific survival. Patients aged <55 years with stage T4N1M0 FVPTC had higher mortality rates per 1,000 person-years than patients in the same stage according to the 8th AJCC System. Cox proportional hazards regression reflected that patients aged <55 years with stage T1-3, any N, M0 or T4N0M0 disease (p=0.001) and patients aged ≥55 years with T1-2N0M0 disease (p=0.004) had signiﬁcantly lower risks of cancer-specific survival (CSS) than those aged <55 years with stage T4N1M0 disease. The CSS curve of patients aged <55 years with stage T4N1M0 disease showed a decline on comparison with others belonging to stage I (p<0.001); and the curve was even not different from patients in stage II and stage III (p>0.05).

**Conclusion:**

Patients aged <55 years with stage T4N1M0 FVPTC had worse survival than patients in stage I; no difference was seen on comparison with stage II patients. We recommend this group of patients be upstaged in the 8th AJCC system.

## Introduction

FVPTC is a major subtype of thyroid cancer, which is the most common endocrine malignancy (1). Some studies have shown that the incidence of thyroid cancer has substantially increased in the last few decades (2, 3). The global thyroid cancer incidence rates have undergone a 3.8‐fold increase since the 1970s (3, 4). Approximately 90% of malignant thyroid tumors involve differentiated thyroid cancer (DTC), and DTCs are classiﬁed as either papillary thyroid carcinoma (PTC) or follicular thyroid carcinoma based on the histologic pattern (5). PTC, as the most frequent type of thyroid malignancy, has two main subtypes: pure papillary thyroid carcinoma and follicular variant of papillary thyroid carcinoma (FVPTC). FVPTC is composed of follicles lined by cells exhibiting nuclear features of PTC (6).

A variety of staging systems have been used to identify different prognostic groups and predict their survival in terms of DTC; among them, the American Joint Committee on Cancer tumor-node-metastasis (AJCC/TNM) cancer staging system is widely used (7). The newest (8th) edition of the TNM classification (TNM-8th), published in 2017, contained some significant changes from the previous edition (8). In this edition, the age cutoff value increased from 45 to 55 years, and the definitions of primary tumor (T) and regional lymph (N) stages were changed from those in the 7th edition; meanwhile, the AJCC prognostic stage groups underwent a series of adjustments (9, 10). As a result, down-staging appeared in nearly one third of DTC patients (11, 12).

Several studies about the new staging system have shown that its predictive value for survival is better than that of the seventh edition (13, 14). However, we don’t know whether the new system predicts the survival of FVPTC patients accurately; only a few studies have evaluated the relationship between FVPTC and the new classification system. Therefore, it is necessary to thoroughly evaluate the correlation between the new system and survival prediction in terms of FVPTC. In this study, we focused on whether each subgroup of different stages aligns with its newest classification and compared the survival in these subgroups.

## Materials and Methods

### Data Collection

The Surveillance, Epidemiology, and End Results (SEER) database of the National Cancer Institute (https://seer.cancer.gov/) was used as the source of data. The steps to access the SEER data were available using the link below.

https://seer.cancer.gov/data/access.html

Since SEER is a publicly available database with anonymized data, no ethical review was required. All patients diagnosed with FVPTC were identified using histopathology codes of the International Classification of Disease for Oncology, third edition (ICD-O-3). The eligible diagnosis was “papillary carcinoma, follicular variant.” There were totally 18,307 FVPTC patients enrolled in the SEER database whose diagnosis year were from 2010 to 2015. We subsequently excluded 654 patients (those with T0, TX, T4NOS, NX, or N1NOS disease; missing data for survival; and two special events). Consequently, 17,662 FVPTC patients were included in this research.

### More Detailed Staging Groups

Patients with FVPTC were divided into Stage I, Stage II, Stage III, and Stage IV based on the TNM-8th system. Then, Stage I patients were further divided into the following groups: age <55 T1-3, any N, M0 and T4N0M0; group age <55 T4N1M0; and group age ≥55 T1-2N0M0. Stage II patients were further divided into the following groups: age <55 any T, any N, M1; and group age ≥55 T1-2N1M0 and T3, any N, M0.

### Statistical Analysis

Quantitative variables were presented as medians (interquartile range) and categorical variables were expressed as number (%). The factors associated with cancer-specific survival (CSS) and overall survival (OS) were identified by Cox regression analyses, respectively. Then, the hazard ratio (HR) and 95% confidence interval (CI) were calculated. We also calculated and compared cancer-specific mortality (CSM) and all-cause mortality (ACM) rates per 1,000 person-years for each subgroup. Cox proportional hazards regression analyses with adjustment for demographic, pathological, and treatment features were performed to quantify the risk of CSS and OS. Finally, survival curves were produced by Kaplan-Meier analyses using log-rank tests. Statistical significance was defined using a two-sided p<0.05. These analyses were performed using SPSS version 22.0 (IBM Corp., Armonk, NY), GraphPad Prism version 8 (GraphPad Software Inc., La Jolla, CA), and Stata/SE version 14 (Stata Corp., College Station, TX).

## Results

### Demographic and Clinical Characteristics

The demographic and clinical characteristics of the 17,662 FVPTC patients are listed in **Table 1**. The 17,662 patients included 14,013 (79.3%) women and 3,649 (20.7%) men. The median age was 50 (interquartile range, 39–60) years. According to the TNM-8th system, 15,992 patients (90.5%) were placed in stage I; 1,457 (8.2%) in stage II; 92 (0.5%) in stage III; and 121 (0.7%) in stage IV. The number of patients in each subgroup is displayed in **Table 2**. There were 10,871 (61.6%) patients aged <55 years with stage T1-3, any N, M0 or T4N0M0. The numbers of patients in the groups age <55 T4N1M0 and age ≥55 T1-2N0M0 were 82 (0.5%) and 5,039 (28.5%), respectively. Regarding Stage II, the subgroups age <55 any T, any N, M1 and age ≥55 T1-2N1M0 or T3, any N, M0 included 52 (0.3%) and 1,405 (8.0%) patients, respectively.

**Table 1:** Demographic and clinicopathological characteristics of 17662 patients with FVPTC.

Characteristics	Number (%)
Age at diagnosis (year)	
Median (interquartile range)	50 (39–60)
Year of diagnosis	
2010–2012	7,912 (44.8)
2013–2015	9,750 (55.2)
Sex	
Female	14,013 (79.3)
Male	3,649 (20.7)
Race	
White	14,197 (81.6)
Black	1,607 (9.2)
Other	1,604 (9.2)
AJCC Staging Grouping (8th Edition)	
Stage at diagnosis I	15,992 (90.5)
Stage at diagnosis II	1,457 (8.2)
Stage at diagnosis III	92 (0.5)
Stage at diagnosis IV	121 (0.7)
T stage at diagnosis	3,649 (20.7)
T1	10,695 (60.6)
T2	3,530 (20.0)
T3	3,147 (17.8)
T4a	209 (1.2)
T4b	81 (0.5)
Lymph node metastasis	2,223 (12.5)
Distant metastasis	138(0.8)
Multifocality	8,227(46.9)
Extrathyroidal extension	1,873 (10.6)
Radiation therapy	
None or refused	9,603(54.4)
Yes	8,059(45.6)
Surgery	
Biopsy	44 (0.3)
Lobectomy	2,672(15.2)
Subtotal or near-total thyroidectomy	394(2.2)
Total thyroidectomy	14,461(82.3)

**Table 2:** Demographic characteristics of each subgroup patients with FVPTC.

AJCC Staging Grouping(8th Edition)	Number (%)
Stage at diagnosis I	15,992 (90.5)
Age <55 T1-3, any N, M0 and T4N0M0	10,871 (61.6)
Age <55 T4N1M0	82 (0.5)
Age ≥55 T1-2N0M0	5,039 (28.5)
Stage at diagnosis II	1,457 (8.2)
Age <55 any T, any N, M1	52 (0.3)
Age ≥55 T1-2N1M0 and T3, any N, M0	1,405 (8.0)
Stage at diagnosis III	
Age ≥55 T4a, any N, M0	92 (0.5)
Stage at diagnosis IV Age ≥55 T4b, any N, M0 and any T, any N, M1	121 (0.7)

### Clinicopathological Factors Associated With CSS

In the univariate Cox regression analysis, age at diagnosis, sex, race, T-stage at diagnosis, lymph node metastasis (LNM), distant metastasis, extrathyroidal extension, and surgery were significant prognostic factors of CSS (all, p < 0.05). Meanwhile, in the multivariate analyses, CSS was associated with age at diagnosis, race, T-stage, distant metastasis, radiation therapy, and surgery (all, p < 0.05). Consequently, LNM may have a combined effect with other factors on CSS (**Table 3**). Cox analyses of the factors associated with OS showed similar results (**Table S1**).

**Table 3:** Clinicopathological parameters associated with the cancer-specific survival.

Parameters		HR	Univariate			HR	Multivariate		
			95% CI		p value		95% CI		p value
Age at diagnosis		1.098	1.075	1.122	<0.001*	1.079	1.055	1.104	<0.001*
Year at diagnosis	2010–2012	ref				ref			
	2013–2015	1.018	0.557	1.858	0.955	0.788	0.406	1.530	0.482
Sex	Female	ref				ref			
	Male	2.499	1.462	4.273	0.001*	1.233	0.670	2.266	0.501
Race	White	ref				ref			
	Black	2.570	1.313	5.027	0.006*	2.162	1.-29	4.542	0.042*
	Other	1.711	0.764	3.832	0.192	0.993	0.409	2.411	0.988
T-Stage at diagnosis	T1	ref				ref			
	T2	2.444	0.656	9.102	0.183	3.441	0.915	12.936	0.067
	T3	4.730	34.315	12.739	<0.001*	10.732	3.299	34.908	<0.001*
	T4a	119.080	41.372	342.746	<0.001*	38.268	8.190	178.808	<0.001*
	T4b	587.565	217.982	1583.770	<0.001*	88.444	19.767	395.724	<0.001*
Lymph node metastasis	No	ref				ref			
	Yes	8.323	4.922	14.074	<0.001*	1.934	0.984	3.804	0.056
Distant metastasis	No	ref				ref			
	Yes	82.539	48.037	141.821	<0.001*	13.225	6.427	27.213	<0.001*
Multifocality	No	ref				ref			
	Yes	1.140	0.669	1.943	0.630	1.121	0.618	2.035	0.707
Extrathyroidal extension	No	ref				ref			
	Yes	20.970	11.692	37.612	<0.001*	1.424	0.530	3.825	0.484
Radiation therapy	None or refused	ref				ref			
	Yes	1.476	0.869	2.506	0.150	0.476	0.245	0.924	0.028*
Surgery	Biopsy	ref				ref			
	Lobectomy	0.016	0.005	0.051	<0.001*	0.169	0.043	0.667	0.011*
	Subtotal or near total thyroidectomy	0.014	0.002	0.124	<0.001*	0.138	0.014	1.406	0.094
	Total thyroidectomy	0.018	0.007	0.045	<0.001*	0.087	0.024	0.317	<0.001*

*represent the p value <0.05.

### CSM and ACM Rates per 1,000 Person-Years

The CSM and ACM rates per 1,000 person-years are shown in **Table 4**. The CSM in the group age <55 T4N1M0 (3.675, 95% CI: 0.518–26.092) was higher than those in the group age <55 T1-3, any N, M0 or T4N0M0 (0.105, 95% CI: 0.039–0.280) and group age ≥55 T1-2N0M0 (0.115, 95% CI: 0.029–0.461), both of which belonged to stage I; moreover, the CSM in the group age <55 T4N1M0 was even higher than that in a subgroup of stage II (age ≥55 T1-2N1M0 or T3, any N, M0) (1.942, 95% CI: 1.010–3.732). The CSM in the group age <55 T4N1M0 was higher than that in the stage I group and was similar to that in the stage II group. The ACM rates showed similar results.

**Table 4:** Measures of cancer-specific mortality and all-cause mortality of FVPTC.

		Total Number	Cancer-specific Mortality	%	Cancer-specific Mortality	95%CI	All-cause Mortality	%	All-cause Mortality	95%CI
			No.		1,000 Person-Years		No.		1,000 Person-Years	
Stage I	Age <55 T1-3, any N, M0 and T4N0M0	10,871	4	0.037	0.105	0.039–0.280	64	0.589	1.549	1.200–1.999
	Age ≥55 T1-2N0M0	5,039	3	0.060	0.115	0.029–0.461	138	2.734	7.557	6.368–8.969
	Age <55 T4N1M0	82	1	1.220	3.675	0.518–26.092	3	3.659	11.026	3.556–34.187
Stage II	Age <55 any T, any N, M1	52	1	1.923	5.55	0.782-39.403	2	3.846	11.101	2.776–44.386
	Age ≥55 T1-2N1M0 and T3, any N, M0	1,405	10	0.712	1.942	1.010–3.732	55	3.915	11.651	8.924–15.213
Stage III	Age ≥55 T4a, any N, M0	92	6	6.522	21.065	9.464–46.888	15	16.304	45.641	26.502–78.602

### Hazard Ratios of Different Subgroups for CSS

The HRs for CSS of the group age <55 T4N1M0 compared with the other groups are displayed in **Table 5**. The unadjusted HR of the group age <55 T1-3, any N, M0 or T4N0M0 was 0.028 (95% CI: 0.003–0.252, p=0.001). The HR adjusted for demographic data was 0.028 (95% CI: 0.003–0.256, p=0.002). The HR adjusted for demographic and pathological data was 0.029 (95% CI: 0.003–0.268, p=0.002). The HR adjusted for demographic, pathological, and therapeutic data was 0.021 (95% CI: 0.002–0.198, p=0.001). As to the group age ≥55 T1-2N0M0, the Cox regression HRs for unadjusted, adjusted 1, adjusted 2, and adjusted 3 models were 0.047 (95% CI: 0.005–0.448, p=0.008), 0.046 (95% CI: 0.005–0.441, p=0.008), 0.048 (95% CI: 0.005–0.469, p=0.009), and 0.033 (95% CI: 0.003–0.345, p=0.004), respectively. Meanwhile, the adjusted P values for the subgroups of stage II and stage III were all more than 0.05. These data indicated that the group age <55 T4N1M0 differed significantly from the subgroups of stage I but not from those of stage II or even stage III. The HRs for OS are displayed in **Table S2**, and they showed similar results.

**Table 5:** Hazard ratios of AJCC Cancer Staging (8th Edition) for cancer-specific survival.

Stage at diagnosis	Stage based on TNM-8th system	Unadjusted Cox regression		Adjusted 1 Cox regression		Adjusted 2 Cox regression		Adjusted 3 Cox regression	
		Hazard Ratio	p-value	Hazard Ratio	p-value	Hazard Ratio	p-value	Hazard Ratio	p-value
		(95% CI)		(95% CI)		(95% CI)		(95% CI)	
Age <55 T4N1M0	I	ref		ref		ref		ref	ref
Age <55 T1-3, any N, M0 and T4N0M0	I	0.028(0.003–0.252)	0.001*	0.028(0.003–0.256)	0.002*	0.029(0.003–0.268)	0.002*	0.021(0.002–0.198)	0.001*
Age ≥55 T1-2N0M0	I	0.047(0.005–0.448)	0.008*	0.046(0.005–0.441)	0.008*	0.048(0.005–0.469)	0.009*	0.033(0.003–0.345)	0.004*
Age <55 any T, any N, M1	II	1.467(0.092–23.463)	0.786	1.421(0.089–22.753)	0.804	1.455(0.091–23.335)	0.791	1.339(0.082–21.993)	0.838
Age ≥55 T1-2N1M0 and T3, any N, M0	II	0.577(0.074–4.504)	0.599	0.557(0.071–4.363)	0.578	0.581(0.074–4.576)	0.606	0.449(0.056–3.607)	0.452
Age ≥55 T4a, any N, M0	III	5.501(0.662–45.703)	0.115	5.628(0.674–46.983)	0.111	0.029(0.003–0.268)	0.002*	4.157(0.475–36.386)	0.198

Adjusted 1 Cox regression: cox regression for year at diagnosis, sex and race matched subtype pairs.

Adjusted 2 Cox regression: cox regression for year at diagnosis, sex, race and multifocality matched subtype pairs.

Adjusted 3 Cox regression: cox regression for age at diagnosis, year at diagnosis, sex, race, multifocality, radiation therapy and surgery matched subtype pairs.

*Represent the p value <0.05.

### Kaplan-Meier Analyses Using Log-Rank Tests

**Figure 1:**
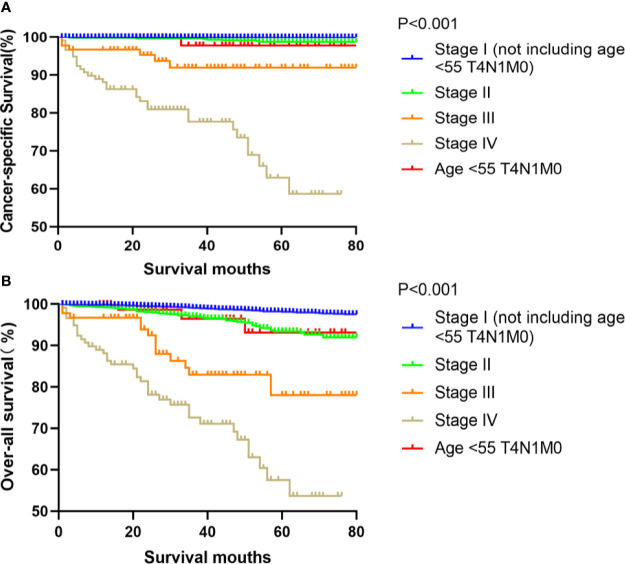
Kaplan–Meier curves for cancer-specific survival **(A)** and overall survival **(B)** between FVPTC patients in stage I (not including age <55 T4N1M0), II, III, IV and those aged <55 years with stage T4N1M0 disease.

**Figure 2:**
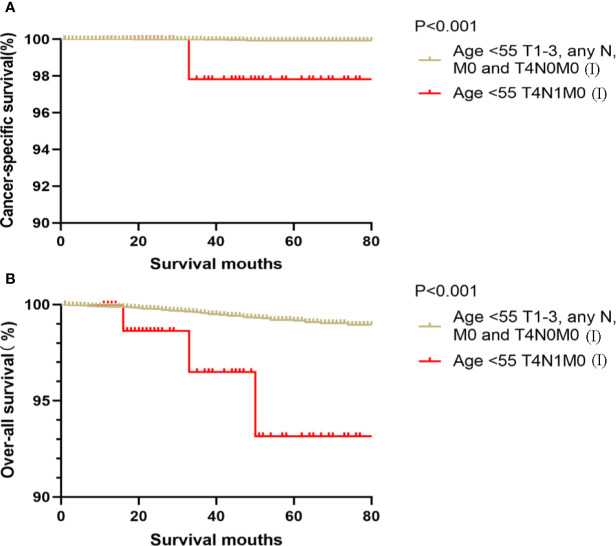
Kaplan–Meier curves for cancer-specific survival **(A)** and overall survival **(B)** between FVPTC patients aged <55 years with stage T1-3, any N, M0 or T4N0M0 disease and patients aged <55 years with stage T4N1M0 disease.

**Figure 3:**
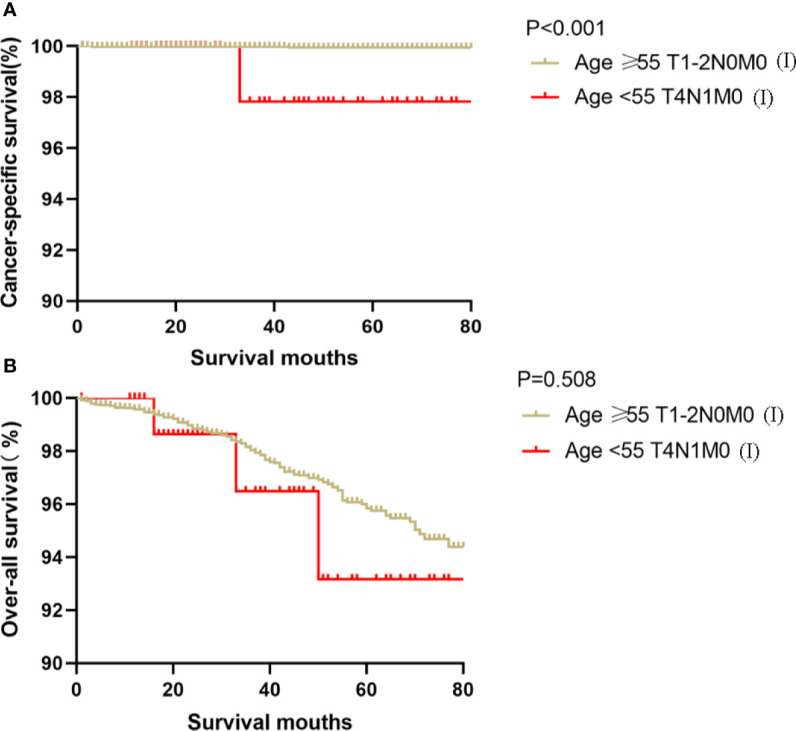
Kaplan–Meier curves for cancer-specific survival **(A)** and overall survival **(B)** between FVPTC patients aged ≥55 years with stage T1-2N0M0 disease and patients aged <55 years with stage T4N1M0 disease.

**Figure 4:**
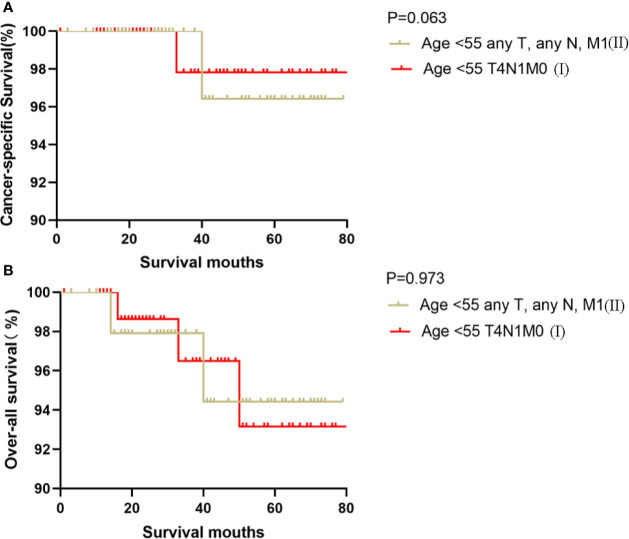
Kaplan–Meier curves for cancer-specific survival **(A)** and overall survival **(B)** between FVPTC patients aged <55 years with stage any T, any N, M1 disease and patients aged <55 years with stage T4N1M0 disease.

**Figure 5:**
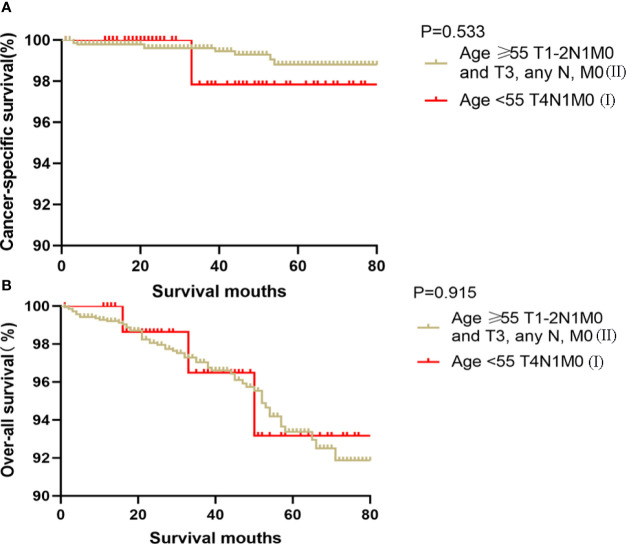
Kaplan–Meier curves for cancer-specific survival **(A)** and overall survival **(B)** between FVPTC patients aged ≥55 years with stage T1-2N1M0 or T3, any N, M0 disease and patients aged <55 years with stage T4N1M0 disease.

**Figure 6:**
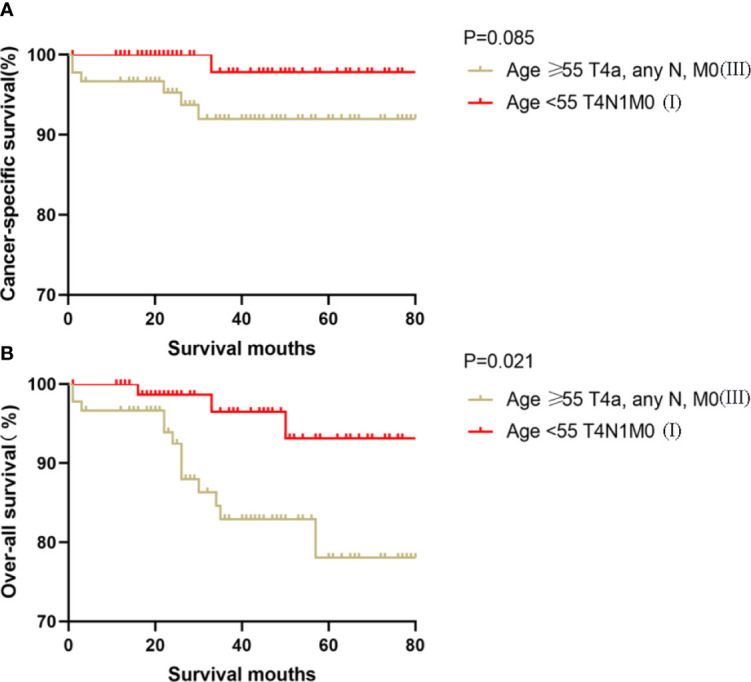
Kaplan–Meier curves for cancer-specific survival **(A)** and overall survival **(B)** between FVPTC patients aged ≥55 years with stage T4a, any N, M0 disease and patients aged <55 years with stage T4N1M0 disease.

Kaplan-Meier analyses using log-rank tests showed that CSS and OS were significantly different between the groups age <55 T4N1M0 and stage I (not including age <55 T4N1M0), stage II, stage III, and stage IV (both, p<0.001). Kaplan–Meier analyses between the group age <55 T4N1M0 and group age <55 T1-3, any N, M0 or T4N0M0 showed significant differences in CSS and OS (both, p <0.001). Meanwhile, compared to the group age ≥55 T1-2N0M0, the group age <55 T4N1M0 showed a significant decline in the CSS curve (p<0.001). Notably, the CSS was not different between the group age <55 T4N1M0 and the subgroups of stage II or even stage III (all, p>0.05). These curves are displayed in **Figures 1**–**6**.

## Discussion

In the eighth edition, some significant changes were made in the age cutoff and the definitions of primary tumor (T) and regional lymph node (N), causing a significant number of patients to be down-staged. Although the new staging system has been shown to have better predictive value for survival prognosis, few studies have focused on the specific effect of the TNM-8th system on FVPTC prognosis. Thus, we explored the applicability of the system in FVPTC patients with the aim of improving its prognosis prediction ability.

In this study, we observed that patients aged <55 years with stage T4N1M0 disease should be upstaged in the TNM-8th cancer staging system. Among the total patients, Stage I, Stage II, Stage III, and Stage IV accounted for 90.5, 8.2, 0.5, and 0.7% of patients according to the TNM-8th system. Each stage was then divided into detailed groups, and it was determined whether these groups conformed to their AJCC/TNM stages. Cox proportional hazard regression analyses revealed that the risk of CSS was higher in the subgroup age <55 T4N1M0 than in the other subgroups in stage I and actually located between subgroups in stage II, by either unadjusted analyses or adjusted analyses. The CSM and ACM rates per 1,000 person-years for this group were higher than those for stage I and similar to those for stage II. Kaplan-Meier curves showed similar results. The results indicated that patients aged <55 years with stage T4N1M0 showed significantly worse survival than patients in other subgroups in stage I and showed no difference in survival rates compared to those in stage II. This suggests that the subgroup age <55 T4N1M0 should not belong to stage I and needs to be upstaged.

We conducted univariate and multivariate Cox proportional hazard regression analyses to identify the prognostic factors associated with CSS and OS. Patients with older age, higher T-stage at diagnosis, LNM, distant metastasis, and extrathyroidal extension can be predicted to have a worse prognosis and require more aggressive treatments such as radiation therapy and surgery. These analyses partly explained why patients aged <55 years with stage T4N1M0 disease had worse survival and needed to be upstaged.

Yan et al. conducted a retrospective study and found that the 10-year mortality rate for PTC increased incrementally without an obvious inflection point according to age groups <35 years to ≥70 years when analyzing age in 5-year increments (15). Similarly, Adam et al. reported a linear association between age and PTC mortality, without an apparent age cutoff demarcating the difference in survival (16). These studies challenged the appropriateness of age cutoffs in staging systems for FVPTC. Moreover, in the eighth edition of the AJCC/TNM system, the cutoff age used for staging was increased from 45 to 55 years. Hence, we speculate that patients aged 45–55 years need a more thorough classification and evaluation, especially those being down-staged.

The results in **Table 3** and **Table S1** indicate that stage T4 is associated with a higher mortality risk than T1-3, in terms of either CSM or ACM. In the AJCC system, primary tumor (T) stage is determined by tumor size and extrathyroidal extension. In the newest system, stage T4 includes gross extrathyroidal extension, and the importance of gross extrathyroidal extension in patients without initial distant metastasis has been further emphasized (17–19). Moreover, Lee et al. expressed the same opinion in their study on PTC patients (20). Hence, patients in stage T4 tend to show poor survival.

LNM is also associated with larger odds of adverse outcomes. Schneider et al. demonstrated that LNM did not independently predict survival in their article (p>0.05) (21). Similarly, in our multivariate Cox analyses, LNM was not an independent prognostic factor for CSS but showed a significant influence in univariate analyses. These findings suggest the existence of a synergic effect. Liu et al. pointed out that the synergic effect of gross extrathyroidal extension beyond the strap muscles (stage T4) and LNM may lead to a worse prognosis in DTC patients (22). Thus, we hypothesized that the worse prognosis of patients aged <55 with T4N1M0 disease may be partly due to the synergic effect of stage T4 and LNM. Based on the above evidence, we hypothesize that the combined effect of gross extrathyroidal extension, LNM, and older age is responsible for the poor prognosis in this patient population.

Our study still has several limitations. Selection bias could not be ruled out due to the retrospective design. Moreover, as FVPTC patients have good prognosis, very few adverse events were noted, and only 82 patients aged <55 years were placed in stage T4N1M0. Hence, further research and more clinical trials need to evaluate this stage among patients with FVPTC.

In conclusion, FVPTC patients aged <55 years in stage T4N1M0 have a worse prognosis than stage I patients and have comparable prognosis compared to stage II patients. Therefore, they should be upstaged and receive a more accurate prognosis prediction. Meanwhile, we recommend more aggressive treatments for older patients with a high T-stage and LNM.

## Data Availability Statement

Publicly available datasets were analyzed in this study. This data can be found here: https://seer.cancer.gov/.

## Author Contributions

LG and ZL provided design of the study. SC, WZe, JH, and YYH organized the database. WL, DH, and LZ performed the statistical analysis. WL wrote the first draft of the manuscript. YHH, ML, WW, CZ, and MW contributed to manuscript revision. All authors read and approved the submitted version.

## Conflict of Interest

The authors declare that the research was conducted in the absence of any commercial or financial relationships that could be construed as a potential conflict of interest.

The handling editor declared a past co-authorship with one the authors ZL.

## References

[1] Zidan J Karen D Stein M Rosenblatt E Basher W Kuten A Pure versus follicular variant of papillary thyroid carcinoma - Clinical features, prognostic factors, treatment, and survival. Cancer (2003) 97(5):1181–5. 10.1002/cncr.11175 12599223

[2] Lim H Devesa SS Sosa JA Check D Kitahara CM Trends in Thyroid Cancer Incidence and Mortality in the United States, 1974-2013. Jama-J Am Med Assoc (2017) 317(13):1338–48. 10.1001/jama.2017.2719 PMC8216772 28362912

[3] Siegel RL Miller KD Jemal A Cancer Statistics, 2018. Ca-Cancer J Clin (2018) 68(1):7–30. 10.3322/caac.21442 29313949

[4] Davies L Welch HG Increasing incidence of thyroid cancer in the United States, 1973-2002. Jama-J Am Med Assoc (2006) 295(18):2164–7. 10.1001/jama.295.18.2164 16684987

[5] Kinder BK Well differentiated thyroid cancer. Curr Opin Oncol (2003) 15(1):71–7. 10.1097/00001622-200301000-00011 12490765

[6] Walts AE Mirocha JM Bose S Follicular variant of papillary thyroid carcinoma (FVPTC): histological features, BRAF V600E mutation, and lymph node status. J Cancer Res Clin Oncol (2015) 141(10):1749–56. 10.1007/s00432-015-1939-9 PMC11823938 25702102

[7] Lamartina L Grani G Arvat E Nervo A Zatelli MC Rossi R 8th edition of the AJCC/TNM staging system of thyroid cancer: what to expect (ITCO#2). Endocr-Relat Cancer (2018) 25(3):L7–L11. 10.1530/erc-17-0453 29192093

[8] Tuttle M Morris L Haugen B Shah J Sosa J Rohren E Thyroid-differentiated and anaplastic carcinoma (Chapter 73). Germany: Springer International Publishing (2017).

[9] Dwamena S Patel N Egan R Stechman M Scott-Coombes D Impact of the change from the seventh to eighth edition of the AJCC TNM classification of malignant tumours and comparison with the MACIS prognostic scoring system in non-medullary thyroid cancer. BJS Open (2019) 3(5):623–8. 10.1002/bjs5.50182 PMC6773661 31592514

[10] Kim K Kim JH Park IS Rho YS Kwon GH Lee DJ The Updated AJCC/TNM Staging System for Papillary Thyroid Cancer (8th Edition): From the Perspective of Genomic Analysis. World JSurg (2018) 42(11):3624–31. 10.1007/s00268-018-4662-2 29750323

[11] Shaha AR Migliacci JC Nixon LJ Wang LY Wong RJ Morris LGT Stage migration with the new American Joint Committee on Cancer (AJCC) staging system (8th edition) for differentiated thyroid cancer. Surgery (2019) 165(1):6–11. 10.1016/j.surg.2018.04.078 30415873 PMC6309303

[12] Nam SH Bae MR Roh JL Gong G Cho KJ Choi SH A comparison of the 7th and 8th editions of the AJCC staging system in terms of predicting recurrence and survival in patients with papillary thyroid carcinoma. Oral Oncol (2018) 87:158–64. 10.1016/j.oraloncology.2018.11.003 30527232

[13] Shteinshnaider M Kalmovich LM Koren S Or K Cantrell D Benbassat C Reassessment of Differentiated Thyroid Cancer Patients Using the Eighth TNM/AJCC Classification System: A Comparative Study. Thyroid (2018) 28(2):201–9. 10.1089/thy.2017.0265 29256827

[14] Kim TH Kim YN Kim HI Park SY Choe JH Kim JH Prognostic value of the eighth edition AJCC TNM classification for differentiated thyroid carcinoma. Oral Oncol (2017) 71:81–6. 10.1016/j.oraloncology.2017.06.004 28688696

[15] Yan H Winchester DJ Prinz RA Wang CH Nakazato Y Moo-Young TA Differences in the Impact of Age on Mortality in Well-Differentiated Thyroid Cancer. Ann Surg Oncol (2018) 25(11):3193–9. 10.1245/s10434-018-6668-2 30039325

[16] Adam MA Thomas S Hyslop T Scheri RP Roman SA Sosa JA Exploring the Relationship Between Patient Age and Cancer-Specific Survival in Papillary Thyroid Cancer: Rethinking Current Staging Systems. J Clin Oncol Off J Am Soc Clin Oncol (2016) 34(36):4415–20. 10.1200/jco.2016.68.9372 PMC6366247 27998233

[17] Perrier ND Brierley JD Tuttle RM Differentiated and Anaplastic Thyroid Carcinoma: Major Changes in the American Joint Committee on Cancer Eighth Edition Cancer Staging Manual. Ca-Cancer J Clin (2018) 68(1):56–63. 10.3322/caac.21439 PMC5766386 29092098

[18] Amin MB Greene FL Edge SB Compton CC Gershenwald JE Brookland RK The Eighth Edition AJCC Cancer Staging Manual: Continuing to build a bridge from a population-based to a more “personalized” approach to cancer staging. Ca-Cancer J Clin (2017) 67(2):93–9. 10.3322/caac.21388 28094848

[19] Casella C Ministrini S Galani A Mastriale F Cappelli C Portolani N The New TNM Staging System for Thyroid Cancer and the Risk of Disease Downstaging. Front Endocrinol (2018) 9:541:541. 10.3389/fendo.2018.00541 PMC6153343 30279679

[20] Lee YK Kim D Shin DY Lee CR Lee EJ Kang S-W The Prognosis of Papillary Thyroid Cancer with Initial Distant Metastasis is Strongly Associated with Extensive Extrathyroidal Extension: A Retrospective Cohort Study. Ann Surg Oncol (2019) 26(7):2200–9. 10.1245/s10434-019-07314-x 30895495

[21] Schneider DF Elfenbein D Lloyd RV Chen H Sippel RS Lymph Node Metastases do not Impact Survival in Follicular Variant Papillary Thyroid Cancer. Ann Surg Oncol (2015) 22(1):158–63. 10.1245/s10434-014-3964-3 PMC4275358 25092163

[22] Liu Z Chen S Huang Y Hu D Wang M Wei W Patients Aged ≥55 Years With Stage T1-2N1M1 Differentiated Thyroid Cancer Should Be Downstaged in the Eighth Edition AJCC/TNM Cancer Staging System. Front Oncol (2019) 9:1093:1093. 10.3389/fonc.2019.01093 31681617 PMC6813624

