# Supplementary material for: Rethinking the 8th AJCC System: Is It Suitable for Patients Aged <55 Years With Stage T4N1M0 Follicular Variant of Papillary Thyroid Carcinoma to Be Placed in Stage I?
Source: Front Oncol. 2020 Dec 11;10:543055. doi: 10.3389/fonc.2020.543055 (PMC7794009; doi:10.3389/fonc.2020.543055)
Supplement: Supplementary file 1 [file DataSheet_1.docx]

Supplement table 1: Clinicopathological parameters associated with the over-all survival

| Parameters | | HR | Univariate | | | HR | Multivariate | | |
| --- | --- | --- | --- | --- | --- | --- | --- | --- | --- |
|  |  |  | 95 % CI | | p value |  | 95 % CI | | p value |
| age at diagnosis | | 1.087 | 1.077 | 1.096 | <0.001* | 1.080 | 1.070 | 1.089 | <0.001* |
| Year at diagnosis | 2010-2012 | ref |  |  |  | ref |  |  |  |
|  | 2013-2015 | 0.804 | 0.614 | 1.051 | 0.111 | 0.704 | 0.534 | 0.929 | 0.013* |
| Sex | Female | ref |  |  |  | ref |  |  |  |
|  | Male | 2.138 | 1.697 | 2.692 | <0.001* | 1.576 | 1.236 | 2.010 | <0.001* |
| Race | White | ref |  |  |  | ref |  |  |  |
|  | Black | 1.546 | 1.118 | 2.136 | 0.008* | 2.039 | 1.493 | 2.785 | <0.001* |
|  | Other | 0.853 | 0.557 | 1.308 | 0.467 | 0.751 | 0.473 | 1.191 | 0.223 |
| T-Stage at diagnosis | T1 | ref |  |  |  | ref |  |  |  |
|  | T2 | 0.681 | 0.479 | 0.968 | 0.032* | 0.947 | 0.659 | 1.360 | 0.768 |
|  | T3 | 1.298 | 0.971 | 1.736 | 0.078 | 1.192 | 0.775 | 1.833 | 0.425 |
|  | T4a | 7.386 | 4.779 | 11.416 | <0.001* | 1.443 | 1.009 | 2.063 | 0.001* |
|  | T4b | 21.293 | 13.529 | 33.514 | <0.001* | 5.858 | 2.778 | 12.350 | <0.001* |
| Lymph node metastasis | No | ref |  |  |  | ref |  |  |  |
|  | Yes | 1.721 | 1.299 | 2.279 | <0.001* | 1.443 | 1.009 | 2.063 | 0.045* |
| Distant metastasis | No | ref |  |  |  | ref |  |  |  |
|  | Yes | 13.433 | 9.052 | 19.934 | <0.001* | 5.723 | 3.522 | 9.299 | <0.001* |
| Multifocality | No | ref |  |  |  | ref |  |  |  |
|  | Yes | 0.828 | 0.661 | 1.036 | 0.099 | 0.928 | 0.730 | 1.179 | 0.541 |
| Extrathyroidal extension | No | ref |  |  |  | ref |  |  |  |
|  | Yes | 2.857 | 2.203 | 3.705 | <0.001* | 1.456 | 0.863 | 2.455 | 0.159 |
| Radiation therapy | None or refused | ref |  |  |  | ref |  |  |  |
|  | Yes | 0.573 | 0.454 | 0.723 | <0.001* | 0.500 | 0.380 | 0.659 | <0.001* |
| Surgery | Biopsy | ref |  |  |  | ref |  |  |  |
|  | Lobectomy | 0.072 | 0.037 | 0.140 | <0.001* | 0.141 | 0.067 | 0.298 | <0.001* |
|  | Subtotal or near total thyroidectomy | 0.047 | 0.018 | 0.124 | <0.001* | 0.095 | 0.034 | 0.266 | <0.001* |
|  | Total thyroidectomy | 0.045 | 0.024 | 0.086 | <0.001* | 0.104 | 0.051 | 0.214 | <0.001* |

Note: * represent the p value <0.05.
