# Supplementary material for: Rethinking the 8th AJCC System: Is It Suitable for Patients Aged <55 Years With Stage T4N1M0 Follicular Variant of Papillary Thyroid Carcinoma to Be Placed in Stage I?
Source: Front Oncol. 2020 Dec 11;10:543055. doi: 10.3389/fonc.2020.543055 (PMC7794009; doi:10.3389/fonc.2020.543055)
Supplement: Supplementary file 2 [file DataSheet_2.docx]

Supplement table 2: Hazard ratios of AJCC Cancer Staging (8th Edition) for over-all survival

| Stage at diagnosis | Unadjusted Cox regression | | Adjusted 1 Cox regression | | Adjusted 2 Cox regression | | Adjusted 3 Cox regression | |
| --- | --- | --- | --- | --- | --- | --- | --- | --- |
|  | Hazard Ratio | p-value | Hazard Ratio | p-value | Hazard Ratio | p-value | Hazard Ratio | p-value |
|  | (95% CI) |  | (95% CI) |  | (95% CI) |  | (95% CI) |  |
| Age <55 T4N1M0 | ref |  | ref |  | ref |  | ref |  |
| Age <55 T1-3, any N, M0 and T4N0M0 | 0.151(0.047-0.479) | 0.001* | 0.171(0.054-0.547) | 0.003* | 0.155(0.048-0.495) | 0.002* | 0.106(0.033-0.340) | <0.001* |
| Age ≥55 T1-2N0M0 | 0.718(0.229-2.255) | 0.571 | 0.795(0.253-2.500) | 0.695 | 0.719(0.228-2.268) | 0.574 | 0.442(0.139-1.408) | 0.167 |
|  |  |  |  |  |  |  |  |  |
| Age <55 any T, any N, M1 | 0.991(0.166-5.929) | 0.992 | 1.029(0.172-6.166) | 0.975 | 0.990(0.165-5.928) | 0.991 | 0.998(0.166-5.982) | 0.998 |
|  |  |  |  |  |  |  |  |  |
| Age ≥55 T1-2N1M0 and T3, any N, M0 | 1.077(0.337-3.442) | 0.901 | 1.118(0.350-3.579) | 0.85 | 1.063(0.332-3.405) | 0.918 | 0.823(0.256-2.646) | 0.744 |
| Age ≥55 T4a, any N, M0 | 4.780(1.384-16.514) | 0.013* | 5.073(1.467-17.542) | 0.010* | 4.464(1.280-15.562) | 0.019* | 4.054(1.161-14.155) | 0.028* |

Adjusted 1 Cox regression: cox regression for year at diagnosis, sex and race matched subtype pairs.

Adjusted 2 Cox regression: cox regression for year at diagnosis, sex, race and multifocality matched subtype pairs.

Adjusted 3 Cox regression: cox regression for year at diagnosis, sex, race, multifocality, radiation therapy and surgery matched subtype pairs.

* represent the p value <0.05.
