# Supplementary material for: Rethinking the 8th AJCC System: Is It Suitable for Patients Aged <55 Years With Stage T4N1M0 Follicular Variant of Papillary Thyroid Carcinoma to Be Placed in Stage I?
Source: Front Oncol. 2020 Dec 11;10:543055. doi: 10.3389/fonc.2020.543055 (PMC7794009; doi:10.3389/fonc.2020.543055)
Supplement: Supplementary file 3 [file DataSheet_3.docx]

Supplement table 3: Mortality of FVPTC patients aged <55 years with stage M0.

| Group | Number | Death | Percentage |
| --- | --- | --- | --- |
| T1N0M0 | 5744 | 1 | 0.02% |
| T1N1M0 | 617 | 0 | 0.00% |
| T2N0M0 | 2199 | 1 | 0.05% |
| T2N1M0 | 272 | 1 | 0.37% |
| T3N0M0 | 1417 | 1 | 0.07% |
| T3N1M0 | 576 | 1 | 0.17% |
| T4N1M0 | 82 | 1 | 1.22% |
